# Exploring Bacteria‐Based Cancer Immunotherapy: Comment on “Discovery of Intratumoral Oncolytic Bacteria Toward Targeted Anticancer Theranostics”

**DOI:** 10.1002/advs.202506869

**Published:** 2025-07-25

**Authors:** Chen‐xi Li, Zhong‐cheng Gong

**Affiliations:** ^1^ Department of Oral and Maxillofacial Oncology and Surgery School/Hospital of Stomatology National Clinical Medical Research Institute Stomatological Research Institute of Xinjiang Uygur Autonomous Region the First Affiliated Hospital of Xinjiang Medical University Urumqi 830054 China; ^2^ Sonnig Biomedical Studio Urumqi 830000 China

A recent study by Goto et al.^[^ ^1^ ^]^ piqued our interest in oncolytic bacteria as anticancer theranostics. In this report, the authors describe a unique tumor‐resident bacteria (*Proteus mirabilis*, PM), combined with natural purple photosynthetic bacteria (*Rhodopseudomonas palustris*, RP), to enhance the anticancer activity. PM is a symbiont with inherent biocompatibility and strong immunogenic oncolytic efficacy; it preferentially grows and proliferates within a targeted milieu, effectively prompting immune cell infiltration of the tumor and triggering strong anticancer responses in various syngeneic mouse models. We commend the authors for the novelty and depth of the work presented. We are particularly interested in the potential for highly targeted immunological elimination of the tumor.

An expansive frontier in biomedicine is emerging via the illumination of the diversity and variability of a plethora of microorganisms, collectively termed microbiota. There is a growing appreciation that ecosystems created by resident bacteria and fungi (the microbiomes) have a profound impact on health and disease.^[^ ^2^ ^]^ For cancer, there is increasing evidence that polymorphic variability in microbiomes between individuals in a population can substantially influence cancer phenotypes.^[^ ^3^, ^4^ ^]^ In the tumor microenvironment, malignant cells are surrounded by a complex network of non‐malignant elements that may have pro‐ or anti‐tumorigenic effects, depending on the cell type and abundance. Tumor‐associated microbiota are an important intrinsic component of the tumor microenvironment in human cancers. Moreover, in vitro and preclinical animal models have indicated that bacteria in the tumor‐associated microbiota play a role in cancer progression, metastasis, immunosurveillance, and radio/chemoresistance. There is strong molecular evidence of intratumoral microbiota across at least 33 major cancer types, and imaging data show the colocalization of pan‐bacterial markers with immune and epithelial cell targets, suggesting that intratumoral bacteria can be intracellular.^[^ ^5^, ^6^, ^7^ ^]^ Tumor‐resident intracellular bacteria, originating from normal adjacent tissues, can be sourced from oral/gut microbiota or other sites (e.g., mucosal organ) via hematogenous spread (**Figure** **1**). However, the biomedical functions of tumor‐resident intracellular bacteria remain unclear.

**Figure 1:**
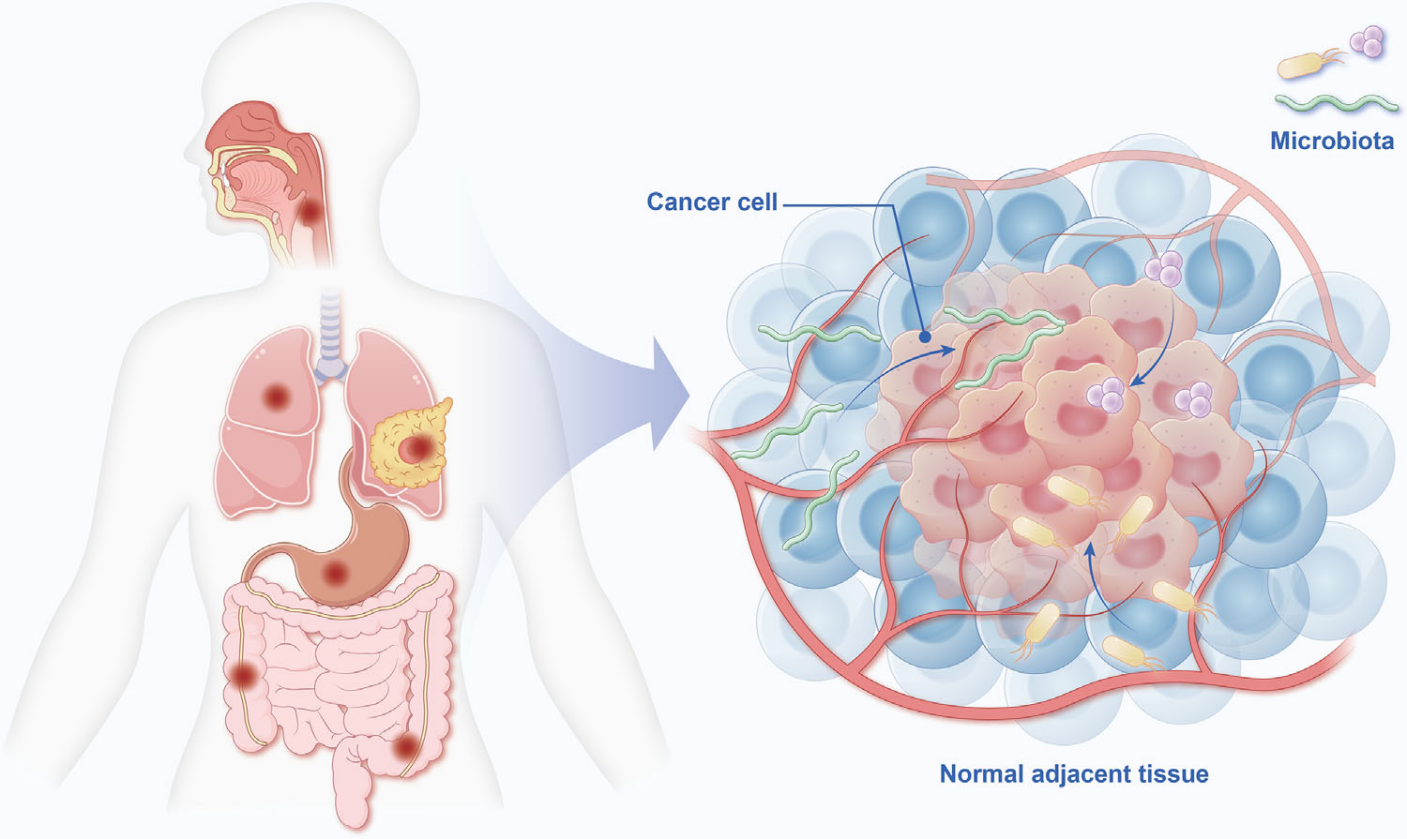
Microbes with complex functions are a potential component in the tumor microenvironment, which serves as a niche for cancer cells and connects cancer to the broader physiology of the host. Intratumor microbes symbiotically associate with the barrier tissues of the body exposed to the external environment—the epidermis and internal mucosa, in particular the oral cavity, gastrointestinal tract, as well as the lung, breast, and urogenital system. Although evidence supports the importance of intratumor microbiota, whether the low‐biomass tumor‐resident microbiota in its physiological homeostatic state plays any significant biological roles in this hypoxic niche has yet to be elucidated.

Immunotherapy has revolutionized cancer treatment but remains constrained by limited response rates, acquired resistance, toxicities, and high costs, necessitating the development of new, innovative strategies. Since the first reported spontaneous regression of tumors in patients with *Streptococcus* spp. In injection (**Figure** **2**),^[^ ^8^ ^]^ cancer biological therapy has evolved into today's immunotherapy over the last century. Although the original strategy was unable to impart maximal therapeutic benefits initially, it laid the foundation for the development of oncolytic bacteria for cancer immunotherapy. Therefore, bacterial immunotherapy emerged as a potent and dynamic strategy for cancer treatment.^[^ ^9^ ^]^ Notably, while the critical importance of bacterial aggregation at tumor sites and the activation of anti‐tumor immunity has long been recognized, its practical application remains elusive. Over the past two decades, remarkable advances have been made in engineering bacteria (either inactivated or viable bacterial strains to stimulate anti‐tumor immunity via tumor‐associated antigens) as agents for cancer immunotherapy, some of which have successfully reached the clinical stages of development.^[^ ^10^, ^11^ ^]^

**Figure 2:**
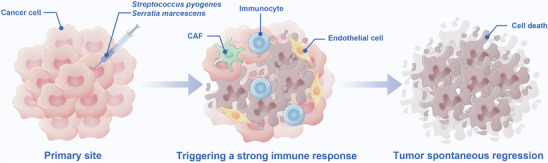
William B. Coley, a pioneering yet under‐recognized figure in early cancer treatment, introduced the concept of using microbial products as immunotherapy for cancer. After learning about a patient with an inoperable malignant neck tumor that disappeared after developing erysipelas, Dr. Coley started treating his patients with cancer, injecting heat‐killed streptococcal organisms in conjunction with *Serratia marcescens* (“Coley's Toxins”) into tumors to treat sarcoma and other malignancies, and observed tumor ablation.^[^ ^8^ ^]^ The use of live bacteria may activate an anti‐tumor immune response through specific tumor‐associated antigens. CAF: cancer‐associated fibroblast.

In this recently published article, the authors aimed to select specially isolated intratumoral bacteria associated with a source of bacteria with high biocompatibility for use in targeted cancer immunotherapy for colorectal cancer, sarcoma, metastatic lung cancer, and extensively drug‐resistant breast cancer models in vivo. The authors initially isolated an extremely effective anticancer bacterium, PM, from a solid tumor in a cell line‐derived xenograft model, such that subcutaneously inoculated colon cancer‐bearing mice could be used for intratumoral oncolytic PM collection. However, this approach is limited to the female mouse model. Jiang et al.^[^ ^12^ ^]^ discovered that estrogen inhibits the growth of colon cancer in mice by reversing the extracellular vesicle‐mediated immunosuppressive tumor microenvironment. The sex distribution among experimental animals should be randomized as much as possible, even though tumor growth in the PBS‐treated group was presented as a control. This randomization approach ensures unbiased allocation while maintaining experimental validity, given the absence of sex‐dependent effects in the authors' preliminary investigations. Equal probability assignment of male and female participants was implemented to eliminate potential confounding variables related to sex differences.^[^ ^13^, ^14^ ^]^ More importantly, the use of subcutaneous tumor implantations rather than orthotopic sites is likely to induce a significant bias, particularly in the field of immunotherapy.^[^ ^15^ ^]^ The tumor in subcutaneous xenografts may alter its growth and dissemination capacities. Additionally, direct orthotopic cell injection between the mucosal and muscularis layers of the cecal wall of nude mice drives tumor foci to more accurately recapitulate the clinical biological behavior of colorectal cancer observed in humans.^[^ ^15^, ^16^ ^]^ Over the past several years, studies have explored intratumoral intracellular microbiota as endogenous physical and biochemical stimuli regulating cancer cell fate decisions, particularly in relation to both physiological and pathological states of disease progression.^[^ ^6^, ^7^, ^17^, ^18^ ^]^

These tumor‐resident microbiotas might be explored to expand upon the list of bacteria assayed in the authors’ screen. Moreover, with the wealth of microbiome and “‐omic” data now available about cancerous lesions both in human patients and animal models, we continue to better understand which gene expression paradigms and extracellular matrix profiles are shared among disease states^[^ ^19^ ^]^ and which are characteristics of specific pathogens or probiotic/oncolytic bacteria. Since pan‐bacterial markers are co‐distributed with both epithelial and immune cell targets, other cell types, including cancer‐associated fibroblasts, may also be investigated to determine the possible functional relationship between microbial presence and host cellular responses within the examined biological system.

Another question that merits further discussion is whether RP tumor‐specific homing occurs. More precise methods for bacterial identification (e.g., colony PCR products for sequencing and sequencing results aligned to the 16S rRNA sequences (Bacteria and Archaea) database in the NCBI BLASTN site) should be carried out not only using tumor tissue but also in other vital organs, such as the heart, liver, spleen, lung, and kidney, after treatment. In addition, to control for false positives due to possible cross‐reactivity of the antibody, quantitative PCR analysis of RP can also be performed.^[^ ^20^ ^]^ It is important to confirm the presence of RP DNA using PCR in samples positive for immunohistochemistry.^[^ ^20^, ^21^ ^]^

Remarkably, the authors demonstrated that the delivery of a bacterial consortium of PM and RP at a ratio of 3:97 via intravenous injection resulted in a complete response after a single dose administration. This finding contrasts with some of the experimental literature, which suggests that an increased abundance of PM plays a key role in colorectal cancer liver metastasis, which is negatively related to Kupffer cell levels.^[^ ^22^ ^]^ Existing publications regarding the impact of PM on cancer immune microenvironments are contradictory.^[^ ^22^, ^23^ ^]^ No definitive references have been cited to explain why intratumoral intracellular PM acts as an oncolytic bacterium in most cancer tissues. Rather than relying solely on pan‐cancer analysis, quantifying and profiling the tissue‐resident microbiome, such as metagenomic sequencing, contamination correction, and antibiotic treatments on tumor cells and mouse models, can help detect the existence of PM in malignancies.

Human association studies and experimental manipulations in mouse cancer models have revealed that particular microorganisms, principally but not exclusively bacteria, can have protective or deleterious effects on cancer development, malignant progression, and response to therapy. Goto et al.^[^ ^1^ ^]^ emphasized that light‐harvesting nanocomplexes of microbial consortia of intratumoral bacteria and purple photosynthetic bacteria can be used to diagnose tumors using a bio‐optical window of near‐infrared light, making them useful theranostic agents for highly targeted immunological elimination of the tumor and precisely marking the tumor location. Intracellular bacterial pathogens harbor elaborate machinery to manipulate host cellular pathways, and whether tumor‐residing bacteria encode similar effectors that enable their survival and dissemination needs to be determined.^[^ ^24^ ^]^ The intratumoral microbiota plays a pivotal role in both cancer pathogenesis and treatment. Particularly, intratumoral bacteria can either promote or inhibit cancer growth by remodeling the tumor immune microenvironment. Achieving a comprehensive understanding of the tumor immune microenvironment is a critical step toward building a mechanistic, organism‐wide model of cancer progression—one that may ultimately unlock the next wave of precision cancer diagnostics and therapeutic innovations.

## Conflict of Interest

The authors declare no conflict of interest.

## Author Contributions

C.L. wrote the original draft and revised the manuscript. Z.G. reviewed the manuscript.
